# Fluorinated Linear Copolyimide Physically Crosslinked with Novel Fluorinated Hyperbranched Polyimide Containing Large Space Volumes for Enhanced Mechanical Properties and UV-Shielding Application

**DOI:** 10.3390/polym12010088

**Published:** 2020-01-03

**Authors:** Qing Li, Ronghua Chen, Yujuan Guo, Fuhou Lei, Zushun Xu, Hui Zhao, Guangfu Liao

**Affiliations:** 1 College of Light Industry and Food Engineering, School of Chemistry and Chemical Engineering, Guangxi University, Nanning 530004, China; liqing@gxun.edu.cn; 2 Guangxi Key Laboratory of Chemistry and Engineering of Forest Products, School of Chemistry and Chemical Engineering, Guangxi University for Nationalities, Nanning 530006, China; c18878733182@163.com (R.C.); G2549857884@163.com (Y.G.); leifuhou@163.com (F.L.); 3 Hubei Collaborative Innovation Center for Advanced Organic Chemical Materials, Ministry of Education Key Laboratory for the Green Preparation and Application of Functional Materials, Hubei University, Wuhan 430062, China; zushunxu@hubu.edu.cn; 4 School of Materials Science and Engineering, Key Laboratory for Polymeric Composite and Functional Materials of Ministry of Education, Guangdong Laboratory of High-Performance Polymer Composites, Sun Yat-sen University, Guangzhou 510275, China

**Keywords:** copolyimide, hyperbranched polyimide, composite, mechanical enhancement, UV-shielding

## Abstract

Fluorinated hyperbranched polyimide (FHBPI), a spherical polymer with large space volumes, was developed to enhance fluorinated linear copolyimide (FPI) in terms of mechanical, UV-shielding, and hydrophobic properties via simple blend and thermal imidization methods. FPI possessed superior compatibility with FHBPI, and no obvious phase separation was found. The incorporation of FHBPI led to the formation of physical crosslinked network between FPI and FHBPI, which markedly improved the mechanical properties of the FPI, resulting in maximum enhancement of 83% in tensile strength from 71.7 Mpa of the pure FPI to 131.4 Mpa of the FPI/FHBPI composite film containing 15 wt % of FHBPI. The introduction of FHBPI also changed the surface properties of composites from hydrophilicity to hydrophobicity, which endowed them with outstanding dielectric stability. Meanwhile, the thin FPI/FHBPI composites kept the high transparency in the visible spectrum, simultaneously showing enhanced UV-shielding properties and lifetimes under intense UV ray. This was attributed to the newly formed charge transfer complex (CTC) between FHBPI and FPI. Moreover, the FPI/FHBPI composites possessed preeminent thermal properties. The properties, mentioned above, gave the composites enormous potential for use as UV-shielding coatings in an environment filled with high temperatures and strong ultraviolet rays.

## 1. Introduction

Known as “gold” in plastics, polyimides (PIs) have excellent properties in many aspects, including mechanical, thermal, low dielectric, optical properties, etc. [1,2,3,4,5,6,7]. Therefore, they are widely used in electronic devices [8,9,10,11,12,13]. However, such mechanical properties still cannot completely meet the requirements in some special fields, such as PI fabrics, aerospace, etc. [14]. As a result, it is quite necessary to strengthen PIs. Many methods have been reported so far. Among them, polymers, inorganic nanoparticles and organic nanoparticles were used to modify PIs.

The incorporation of polymers, e.g., polyurethane (PU), epoxy, etc., could significantly change the mechanical property of PIs. Nevertheless, due to the relative flexibility of their molecular chains, the addition of polymers will not substantially improve the tensile strength, but bring about other improvements, such as flexibility, adhesion, etc. Moreover, the resulting composites often exhibited phase separation, resulting in significant decreases in comprehensive performance [15,16,17]. As strong synergistic effects, many PI/inorganic nanocomposites with enhanced mechanical properties attracted the attention of researchers and were prepared by intercalation, sol-gel processing, and mechanical blend [18,19,20,21,22,23,24,25,26]. Whereas, due to the poor interfacial compatibility, the added amount of inorganic nanoparticles is very critical. Too much can damage the mechanical properties of the composites. In view of those, researches on improving the compatibility of inorganic nanoparticles, with polyimide (PI) matrix has emerged, mainly focused on the surface modification of inorganic nanoparticles. These inorganic nanoparticles, including graphene, graphene oxide, silica, carbon nanotube, zeolite and attapulgite, were modified by silane coupling agents, diisocyanate, silsesquioxane, etc., and then added to the PI matrix. The obtained PI nanocomposite films exhibited significant enhancements in comprehensive performance [27,28,29,30,31,32]. However, not all nanoparticles could be successfully modified during the modification process. The grafting rate of surface modifiers on inorganic nanoparticles was generally low. In consequence, there still existed poor compatibility between the surface-modified inorganic nanoparticles and PI matrix, resulting in some defects at their interface. In addition to polymers and inorganic nanoparticles, some organic nanoparticles were also used as fillers to enhance PI, such as sepia eumelanin. By the addition of amine-modified sepia eumelanin to the PI, 22% and 505% enhancements for tensile strength and elongation at break, respectively, were obtained, indicating that the organic nanoparticles had a significant toughening effect on PI, but the enhancement effect was less obvious. This was because sepia eumelanin was also composed of relatively flexible molecular chains [33]. Moreover, poor interface compatibility existed between amine-functionalized sepia eumelanin and PI. In short, poor compatibility will always be encountered by using the above three methods. More importantly, the enhancement in the tensile strength of PIs was not large enough. Reinforcing materials with good compatibility with PI should be further explored.

Hyperbranched polymers have become one of the research hotspots, due to their special structure, such as many reactive terminal functional groups [34]. Hyperbranched polyimide (HBPI) is one of the most important hyperbranched polymers. Various HBPIs have been successively prepared over the past decades by researchers. They retain some excellent properties of linear polyimides (high-temperature stabilities), and some new features (good solubility) are given [35,36,37]. Different from linear PI molecules, HBPI molecules possess a spherical structure. This structure brings more cavities to the matrix, which lays a foundation for use as gas separation materials [38]. Moreover, HBPI can be applied to the field of optics by modification of terminal functional groups [39,40]. However, it is this three-dimensional spherical structure that makes HBPI poor mechanical properties, due to the lack of physical entanglements between macromolecules. It was reported that increasing physical entanglement between chains could bring about enhanced mechanical strength [41]. In addition, due to the presence of many benzene rings in the HBPI molecule, a large number of charge transfer complex (CTC) can be formed. CTC can absorb a lot of ultraviolet lights, and thus, HBPI has stronger UV absorption performance than linear PI. Similarly, many UV-absorbing polymers have been reported, such as polypyrrole-based composites [42]. They were added into the polymers to enhance the UV absorption properties of the polymers effectively. 

Inspired by these, HBPI with few physical entanglements was incorporated into the liner PI with abundant physical entanglements. Linear PI molecules can just be inserted into the cavities in the molecular structure of HBPI, resulting in the formation of a physical cross-linking network. The PI/HBPI composites will show an improvement on comprehensive properties, including mechanical and UV-shielding properties, due to the strong synergistic effects between liner PI molecules (flexibility and ductility) and HBPI molecules (rigidity and hydrophobicity). It is worth noting that both liner PI and HBPI belong to the PI category. Hence, linear PI and HBPI can be completely integrated with each other, and there is no need to worry about the occurrence of phase separation. The blend of HBPI and linear PI can enhance the mechanical properties of PI and ameliorate the brittleness of HBPI significantly. Currently, few related reports have been proposed. In our previous work, we have synthesized a series of fluorinated hyperbranched polyimide (FHBPI) based on our homemade fluorinated aromatic triamine 1,3,5-tris (4-(2-trifluoromethyl-4-aminophenoxy) phenyl) benzene (TTFAPOPB) and preliminarily studied their mechanical properties [43]. Gel was often formed when synthesizing hyperbranched polyimide. Even if the gel was effectively avoided, each synthesized hyperbranched polyimide was different. Hence, in order to ensure the consistency of FHBPI in composites at each time, large quantities of fluorinated hyperbranched polyamic acid (FHBPAA) were synthesized at one time to spare. In this article, we adopted different ratios of FHBPAA to blend with fluorinated linear copolyimide (FPI), then prepared a series of FPI/FHBPI composites with improved mechanical properties via the physical entanglement and subsequent thermal imidization. Meanwhile, through the choice of fluoromonomers, we supposed to prepare composites with better optical transmission and extend their application in the field of UV shielding. Therefore, the UV-shielding performance of FPI/FHBPI composites was also studied. By the above design, we aim to obtain multifunctional FPI/FHBPI composites with significantly enhanced mechanical, UV-shielding and hydrophobic properties by simple blend method.

## 2. Materials and Methods

### 2.1. Materials

4,4′-Diaminodiphenyl ether (ODA) (98%), 2,2-bis[4-(3,4-dicarboxyphenoxy)phenyl]propane dianhydride (BPADA), 4,4′-(hexafluoroisopropylidene)diphthalic anhydride (6FDA, 98%) were purchased from Energy Chemical (Saan Chemical Technology Co., Ltd., Shanghai, China). 1,3,5-tris(4-(2-trifluoromethyl-4-aminophenoxy)phenyl) benzene (TTFAPOPB) was synthesized by ourselves. Methylene blue trihydrate (BS), TiO_2_ (99.8% metals basis, 25 nm), *N*-methyl-2-pyrrolidone (NMP), and *N*,*N*-dimethylacetamide (DMAC) were purchased from Tokyo Chemical Industry (TCI Development Co., Ltd., Shanghai, China).

### 2.2. Synthesis of Anhydride-Terminated Fluorinated Copolyimide (FPI)

**Scheme 1:**
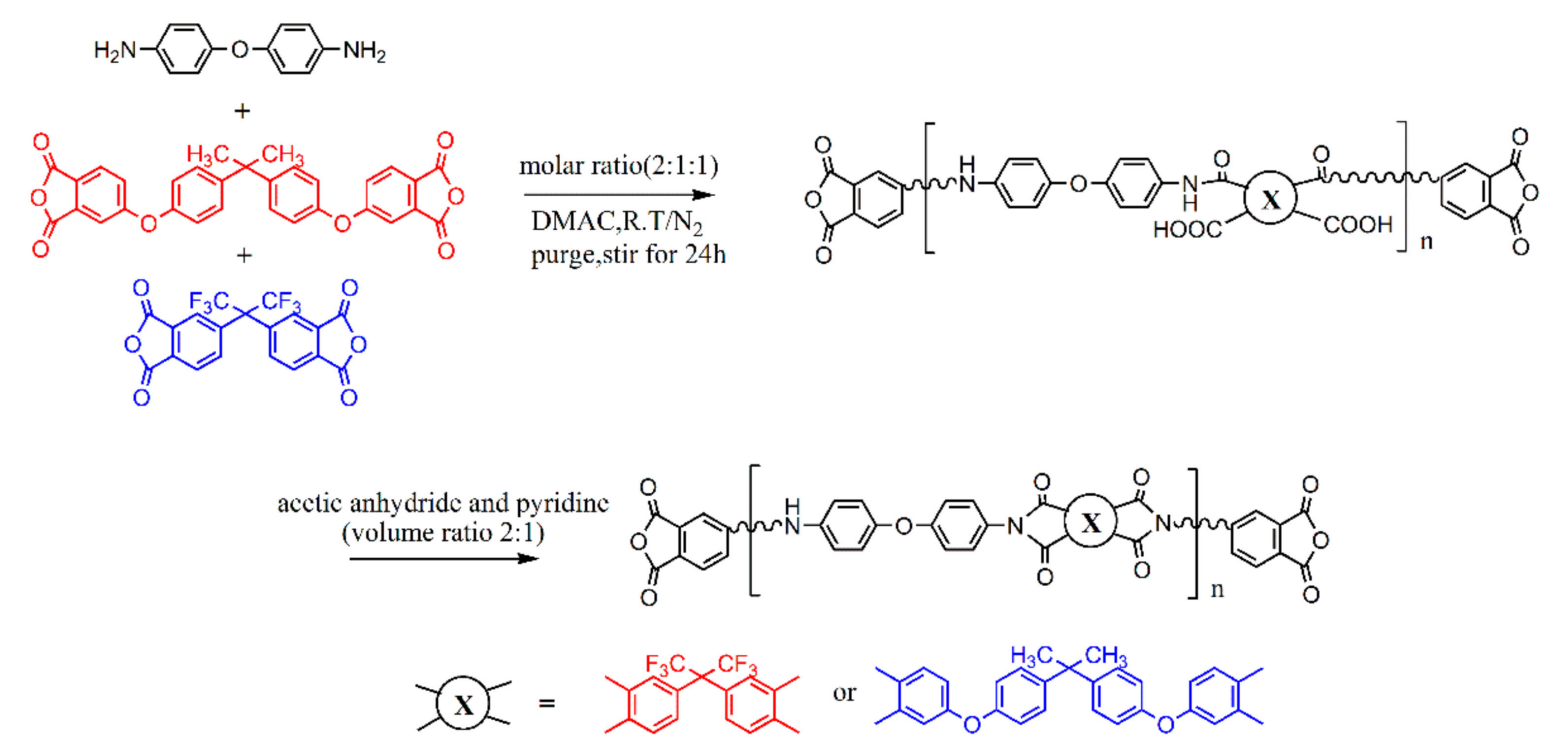
The synthesis process of anhydride-terminated fluorinated linear copolyimide (FPI).

BPADA and 6FDA were used as anhydride sources, and ODA was utilized as an amine source. The FPI was prepared via the following procedures (Scheme 1). Typically, 6 mmol ODA and 38 mL DMAC were put into a 100 mL flask under magnetic stirring. After the complete dissolution of ODA, 3.06 mmol BPADA and 3.06 mmol 6FDA were subsequently added to the flask. The reaction was continued for 24 h at room temperature under N_2_ atmosphere. Then, a fluorinated polyamic acid (PAA) solution was afforded. Afterwards, 0.3 mmol BPADA was replenished to gain anhydride-terminated PAA solution. Then, through chemical imidization to convert PAA to PI, in brief, 7.2 mL acetic anhydride and 3.6 mL pyridine were added to the PAA solution under stirring for 18 h at room temperature and then heating at 60 °C for another 6 h. The obtained solution was slowly added to ethanol with flocculent solid precipitated, which was washed several times with ethanol and dried at 100 °C for 24 h.

### 2.3. Preparation of Anhydride-Terminated FHBPAA

**Scheme 2:**
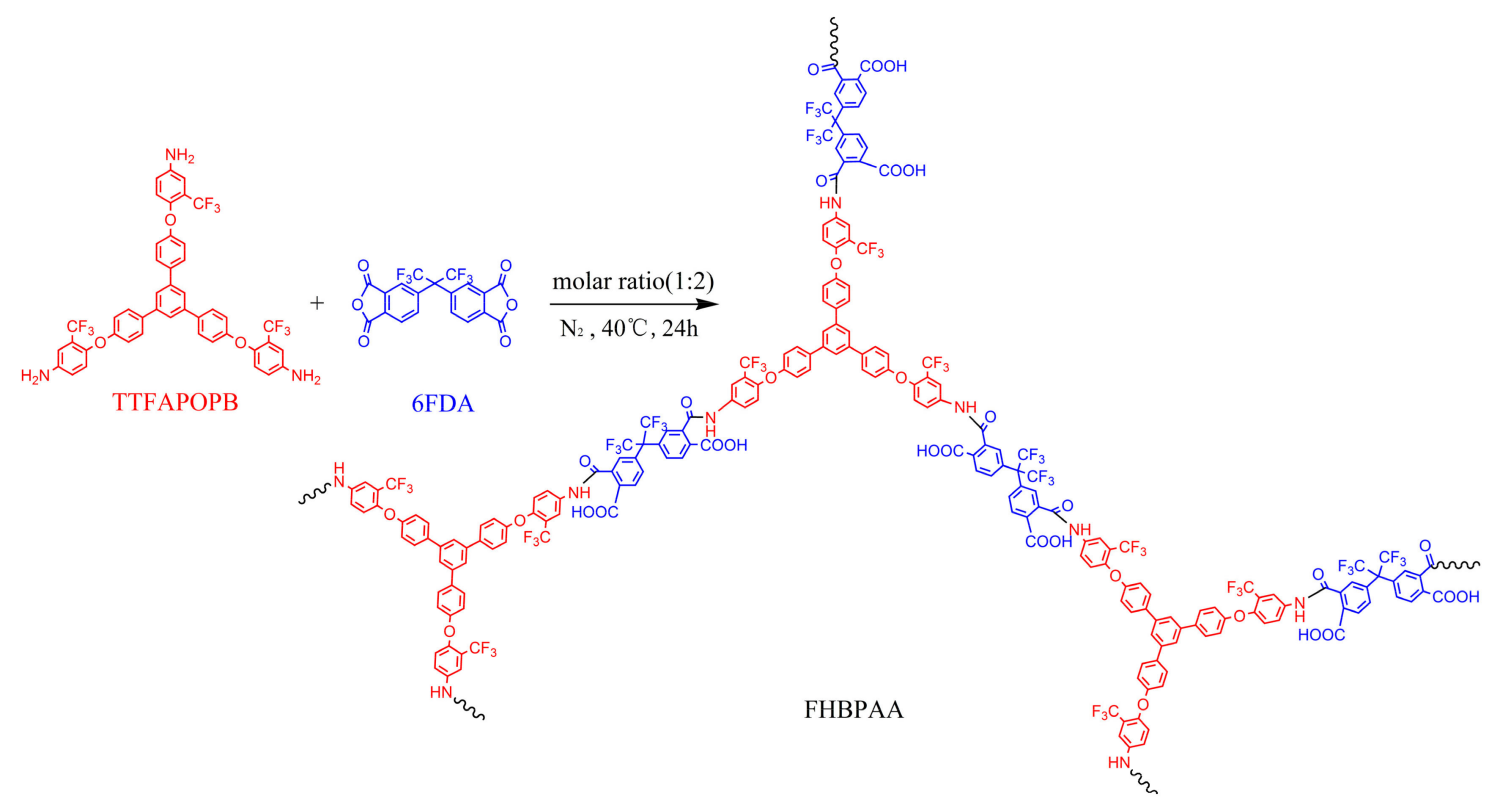
The synthesis process of the FHBPAA. FHBPAA: Fluorinated hyperbranched polyamic acid.

The anhydride-terminated FHBPAA was prepared via the following procedures (Scheme 2). Concretely, 1 mmol 6FDA was dissolved in 10 mL NMP under N_2_ at 40 °C. The solution containing TTFPOPB (0.5 mmol) and NMP (10 mL) was then added dropwise into the above solution for 4 h, affording the anhydride-terminated FHBPAA solution after extra stirring for 24 h. Then store it into the refrigerator to spare.

### 2.4. Preparation of Fluorinated Linear Copolyimide/Fluorinated Hyperbranched Polyimide (FPI/FHBPI) Composites

**Scheme 3:**
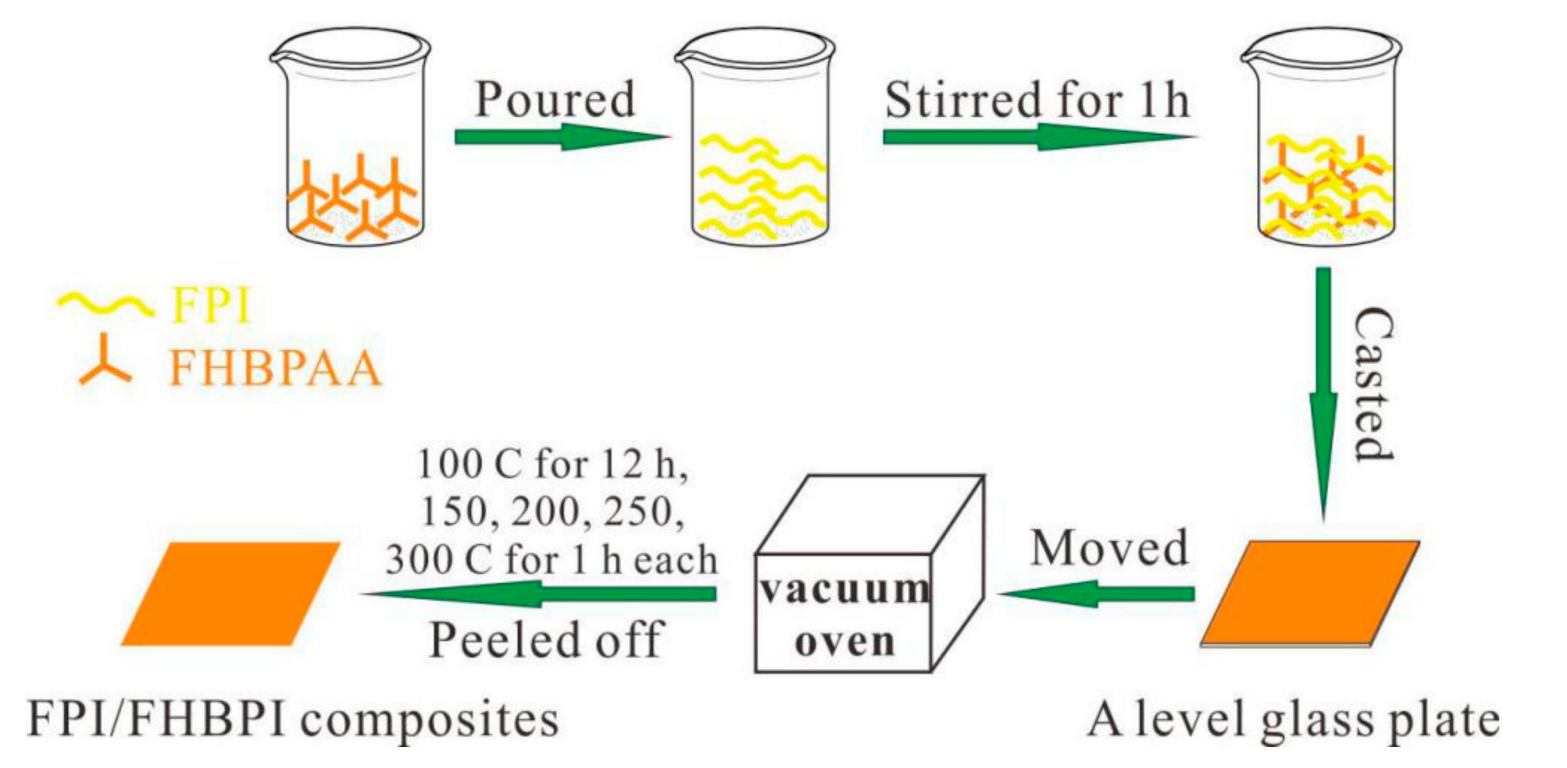
Schematic illustrations of the preparation of FPI/ fluorinated hyperbranched polyimide (FHBPI) composites.

Schematic diagram of preparing FPI/FHBPI composites was shown in Scheme 3. Typically, a certain proportion of FPI and FHBPAA were weighed and mixed in the presence of solvent NMP, thereinto, m(FHBPAA)/m(FPI) = 0%, 5%, 10%, 15%, 20%, 25%, ∞, and the total weight of the two components was set to 0.5 g to ensure that each film was around 0.5 g. The mixture was stirred for one hour, and then poured onto a glass plate with a silicone mold. The curing process was then carried out in a vacuum oven with a stepped temperature program (80 °C for 12 h, 100, 150, 200, 250, 300 °C for 1 h each). The obtained films were cooled down naturally and peeled off, which were 200 ± 20 μm thick, referred to as FPI, FPI/FHBPI-5%, FPI/FHBPI-10%, FPI/FHBPI-15%, FPI/FHBPI-20%, FPI/FHBPI-25%, FHBPI based on the relative content of FHBPAA.

### 2.5. Characterization

Fourier transform infrared spectroscopy (FTIR) analysis of the samples was taken on a Spectrum One FTIR spectrometer (Perkin-Bhaskar-Elmer Co., Waltham, MA, USA). Section morphologies of FPI/FHBPI composites were determined by field emission scanning electron microscopy (FESEM, SUPRA 55, Carl Zeiss AG, Heidenheim, Germany). Thermogravimetric analysis (TGA) was performed using a thermal gravimetric analyzer (STA 449F3Jupiter, Netzsch, Selb, Germany) at a heating rate of 20 °C/min from 30 to 800 °C under N_2_ atmosphere. The dynamic thermomechanical analysis (DMA) was conducted with a Q800 DMA (TA Instruments, New Castle, PA, USA) at 1 Hz, in the temperature range from 150 to about 300 °C, at a heating rate of 5 °C min^−1^ in air. The mechanical properties were performed by stress/strain measure under uniaxial tension (5 mm/min) using a universal testing machine (CMT, SANS, Shenzhen Sans Material Test Instrument Co., Ltd., Shenzhen, China). Surface contact angle (SCA) test was performed on JC2000 D instrument (Shanghai Zhongchen Digital Technic Apparatus Co. Ltd., Shanghai, China). UV-vis spectra were measured with a Mapada UV-1800 spectrometer (Shanghai Mapada Instrument Co., Ltd., Shanghai, China) in the transmittance mode. Water absorption (*WA*) was measured by immersing dried films into deionized water for 24 h at room temperature, then weighing them after fast drying surface moistures, calculated as follows:

$$WA=\frac{Ws-Wd}{Wd}\times 100\%,$$

where *W_d_* and *W_s_* were, respectively, the qualities of the dried film and the swollen one.

The UV-shielding performance of films was evaluated by Methylene blue (MB) degradation experiments in the case of nano-TiO_2_ as a catalyst under a high-pressure mercury lamp (400 W). Concretely, nano-TiO_2_ (10 mg) and MB solution (0.02 mM, 20 mL) were added to a vial wrapped in foil. The dispersion liquid reached adsorption-desorption equilibrium after constantly stirring in the dark for 40 min. The distance between the bottle mouth and UV lamp was set to 15 cm. The whole degradation experiment was carried out in magnetic stirring. Firstly, we set a blank control study without the shielding of any film. In the experimental group, FPI and FPI-FHBPI-15% covered the mouth of the vial in turn. At the appointed time (t), 6 mL of dispersion liquid was collected and centrifuged to remove nano-TiO_2_. The ultraviolet absorption of MB solution at 665 nm was recorded by a Mapada UV-1800 spectrometer. The UV-shielding performance was evaluated using the following formula (*I* = *A*_t_/*A*_0_ × 100%), thereinto, *A*_0_ and *A*_t_ referred to the initial absorbance of MB solution after adsorption-desorption equilibrium and the absorbance of the remaining MB solution covered with the film under UV radiation, respectively.

## 3. Results and Discussion

### 3.1. Characterization of FPI, FPI/FHBPI Composites and FHBPI

A series of FPI/FHBPI composites were prepared through the blending of FPI and FHBPAA and succedent thermal imidization. The FPI was obtained by the polycondensation between ODA, BPADA and 6FDA and chemical imidization. The reason for choosing chemical imidization to prepare FPI was to ensure that the FPI in the composite system was exactly the same. Then, a prescribed amount of configured FHBPAA solution was added to a FPI solution. By thermal imidization, the target products were got. In order to improve the solubility of the FPI and speed up the preparation of the FPI/FHBPI composites, copolymerized fluorinated polyimide (co-FPI) was applied as a substrate.

### 3.2. Structure Analysis

**Scheme 4:**
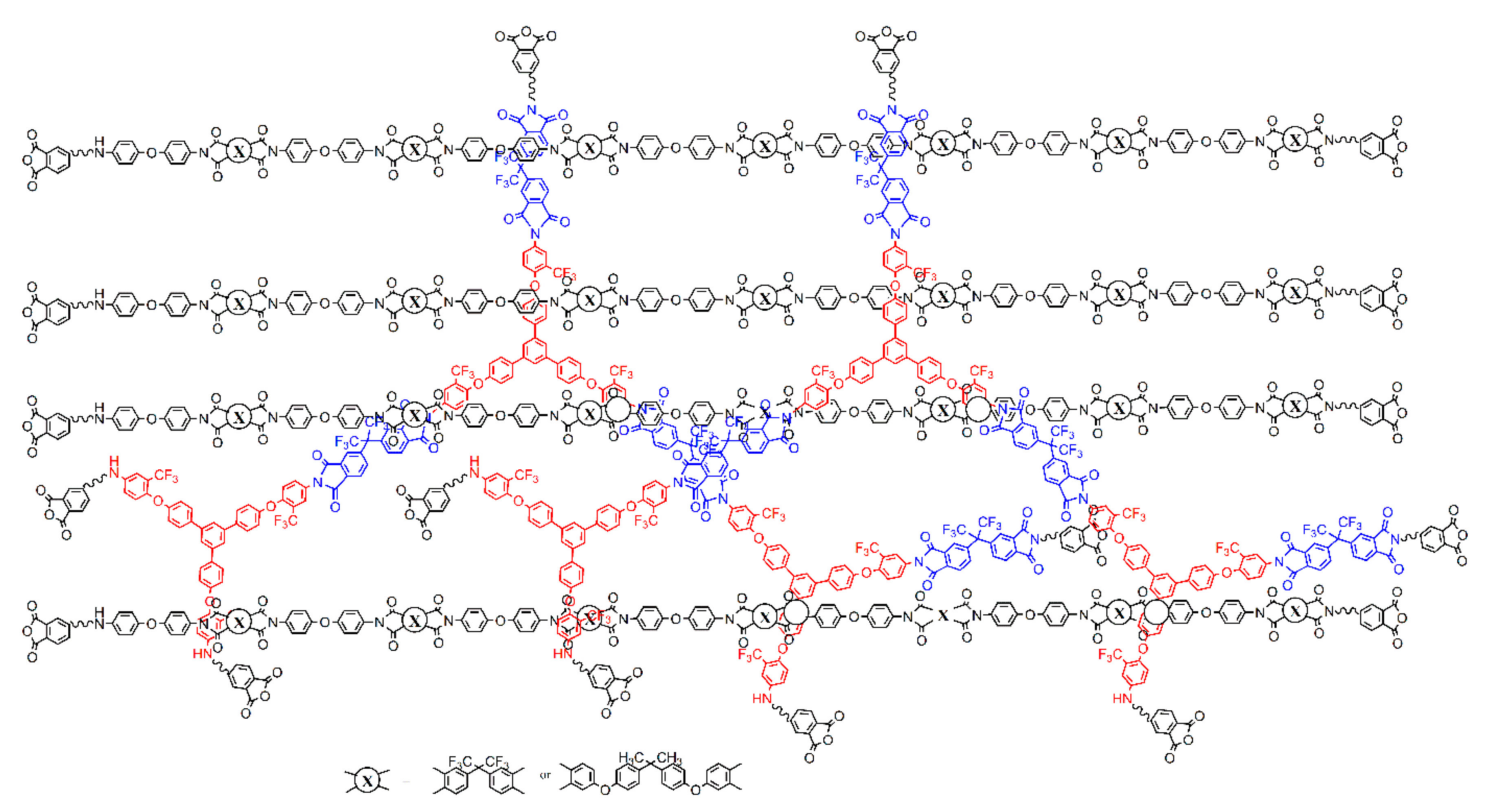
Structural schematic diagram of FPI/FHBPI composites.

**Figure 1:**
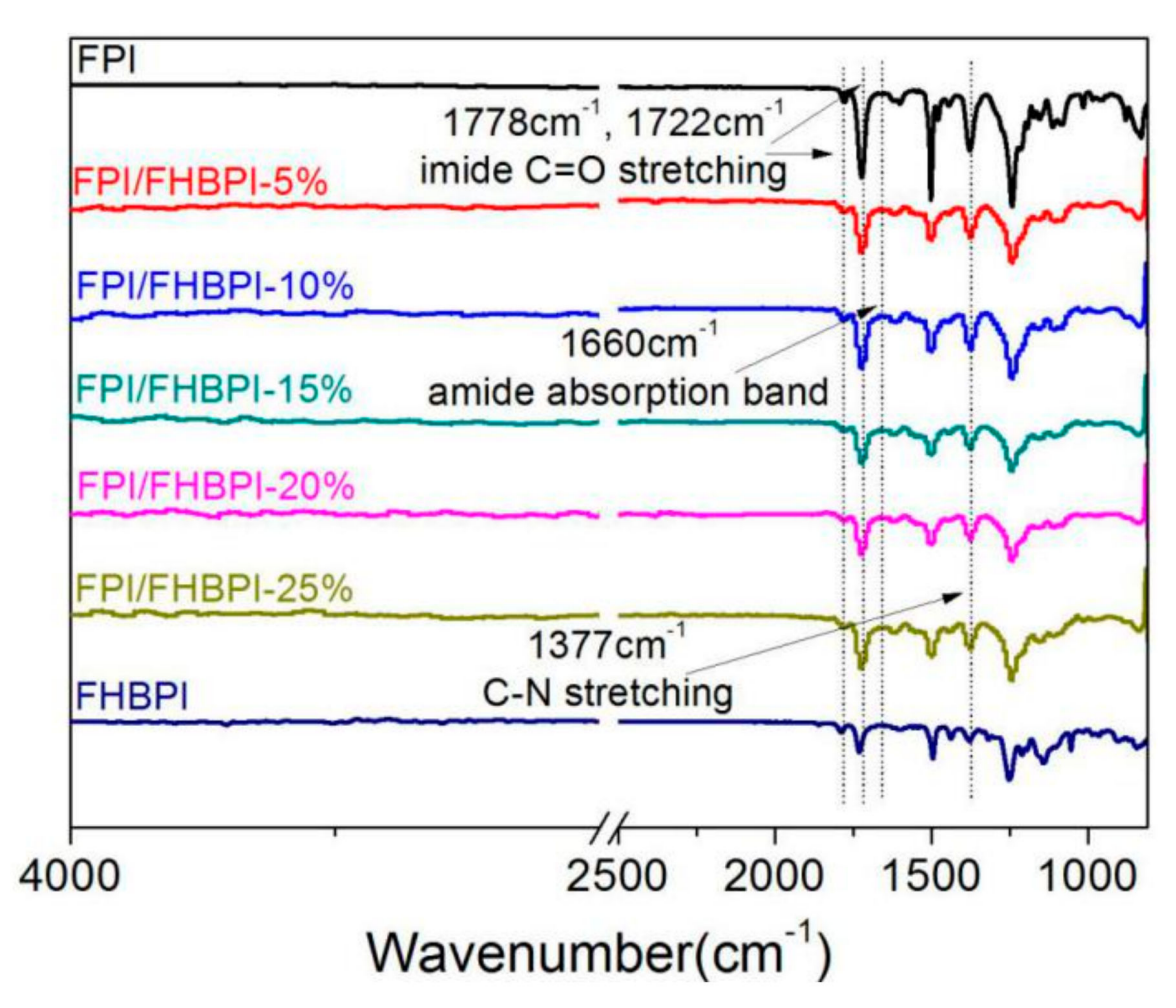
FTIR spectra of FPI, FPI/FHBPI composites and FHBPI.

Structural schematic diagram of FPI/FHBPI composites is displayed in Scheme 4. FHBPI molecules are interspersed with FPI molecules, acting as physical intersection points. Moreover, as shown in Figure 1, the asymmetric stretching and symmetric stretching vibration of C=O appear at 1778 and 1722 cm^−1^ in all of the curves, respectively; and the stretching vibration of C–N also appear at 1376 cm^−1^. Moreover, there is no band at 1660 cm^−1^, attributed to the characteristic absorption of the amide band, demonstrating the complete conversion from PAA to PI.

### 3.3. Thermal Properties

**Figure 2:**
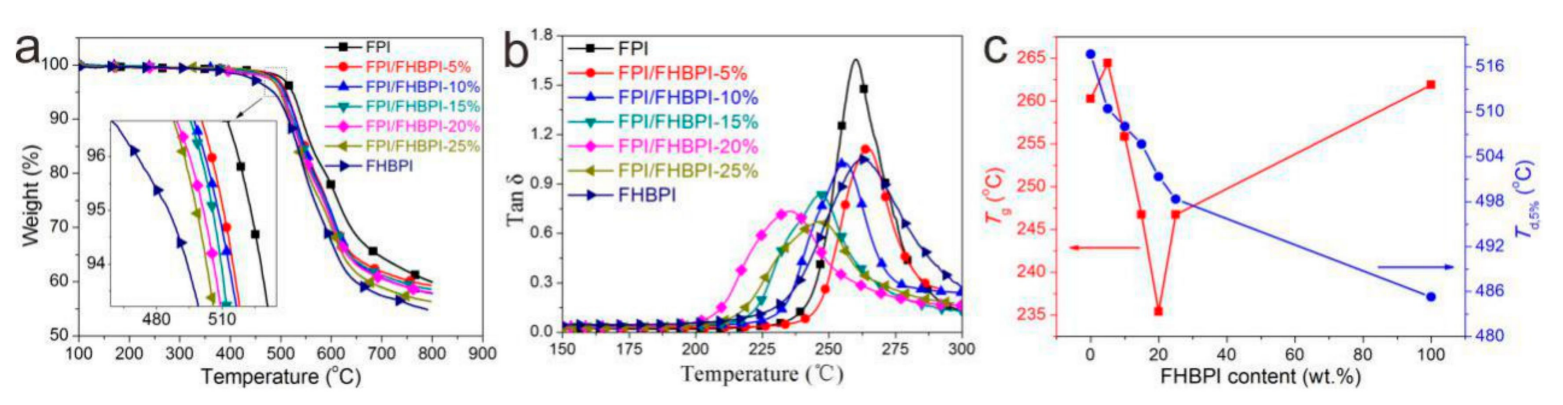
Thermal properties of FPI, FPI/FHBPI composites and FHBPI: (**a**) Thermogravimetric analysis (TGA) curves in N_2_; (**b**) DMA measurement of tanδ-temperature curves; (**c**) Effects of FHBPI content on *T*_d,5%_ and *T*_g_ of PI composites.

Among engineering plastics, polyimides have outstanding thermal stability. Due to their superior thermal stability, they are irreplaceable in numerous fields, such as aerospace, military, etc. The metric indexes, such as 5% decomposition temperature (*T*_d,5%_), 10% decomposition temperature (*T*_d,10%_) and residue weight fraction at 800 °C in N_2_ (*R*_w_) are usually adopted to evaluate the thermal stability of materials, summarized in Table 1. As can be seen from Figure 2a, all the curves had no obvious weight loss before 400 °C, pointing to outstanding thermal stability. Moreover, FPI had the best thermal stability in all films. However, as the FHBPI content increased, the thermal stability of the obtained FPI/FHBPI composites gradually declined, but still superior to pure FHBPI film. The trend was mainly attributed to the hyperbranched structure of FHBPI, which possessed more erratic terminal anhydride than FPI. In addition, *T*_d,5%_ and *T*_d,10%_ of FPI/FHBPI-25% can still be as high as 498.3 °C and 516.2 °C, respectively (Figure 2c). This laid the foundation for their use as heat resistant materials.

In general, the glass transition temperature (*T*_g_) determines the upper limit temperature for polymer materials. It is worth noting that there exists only one peak in the tanδ curve of each FPI/FHBPI composite (Figure 2b), indicating superior compatibility between FPI and FHBPI. The *T*_g_ values first increased, then decreased and increased again with increasing contents of FHBPI. FPI/FHBPI-5% and FPI/FHBPI-20% acquired the maximum *T*_g_ value (264.5 °C), and the minimum one (235.4 °C), respectively. The variation in the *T*_g_ values was clearly described in Figure 2c, which was related to the difficulty in moving molecular segments. When the amount of FHBPI was low (≤5 wt %), FHBPI improved entanglements between FPI molecular chains as a physical cross-linking agent. These pinioned FPI segments were harder to move and relaxed at a higher temperature, leading to an increase of the *T*_g_ value. Nevertheless, when the content of FHBPI was more than 5%, not only FHBPI acted as a physical cross-linking agent, but also its bulky structure played a role. Hyperbranched bulk structure increased the distances between FPI molecular chains, which brought about greater impact than physical cross-linking. Therefore, FPI segments were easier to move, which caused a decrease in *T*_g_ values. When the content of FHBPI continued to increase (≥15 wt %), more FPI molecular segments were immobilized by FHBPI molecules through physical cross-linking, and the free volumes between the FPI molecular chains were also occupied by newly added FHBPI molecules. This was the reason for the reverse increase in *T*_g_s of FPI/FHBPI composites. In summary, the *T*_g_s of the prepared FPI/FHBPI composites were still relatively high, which can meet the basic requirements for use as heat resistant materials.

### 3.4. Strain-Stress Properties

**Figure 3:**
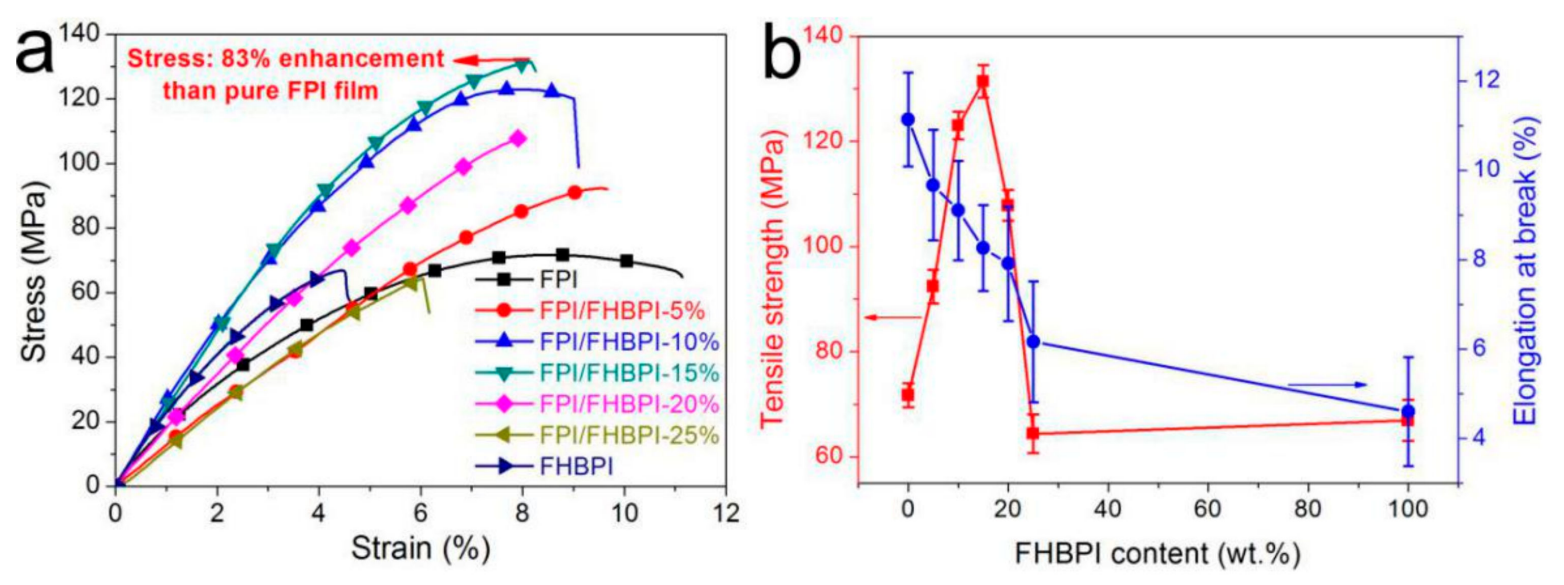
(**a**) Tensile testing curves of FPI, FPI/FHBPI composites and FHBPI; (**b**) Effects of FHBPI content on tensile strength and elongation at break of FPI composite films.

The average tensile strength (σ), and elongation at break (ε_max_) are listed in Table 2. The FPI/FHBPI composites show different mechanical properties with different contents of FHBPI (Figure 3a). The FPI film just fractured after yielding. With the increasing content of FHBPI (≤10 wt %), FPI/FHBPI composites possessed enhanced σ and reduced ε_max_. Meanwhile, all of them had a slight yield phenomenon. Keeping adding FHBPI (15 wt %), it can be seen that yield phenomenon disappeared, and brittle fracture occurred at the elastic deformation of the film. Σ and ε_max_ of FPI/FHBPI-15% reached 131.4 Mpa and 8.26%, respectively. This was the maximum value of σ, which was 1.83 times as much as pure FPI. However, with a further increase (≥20 wt %), σ of the FPI/FHBPI composites started to decline. Effects of FHBPI loading on σ and ε_max_ of FPI/FHBPI composites were summarized in Figure 3b. The addition of FHBPI made σ first increase and then decreased, reaching the maximum at 15 wt % of FHBPI. Meanwhile, ε_max_ of FPI/FHBPI composites had been decreasing with the addition of FHBPI.

**Figure 4:**
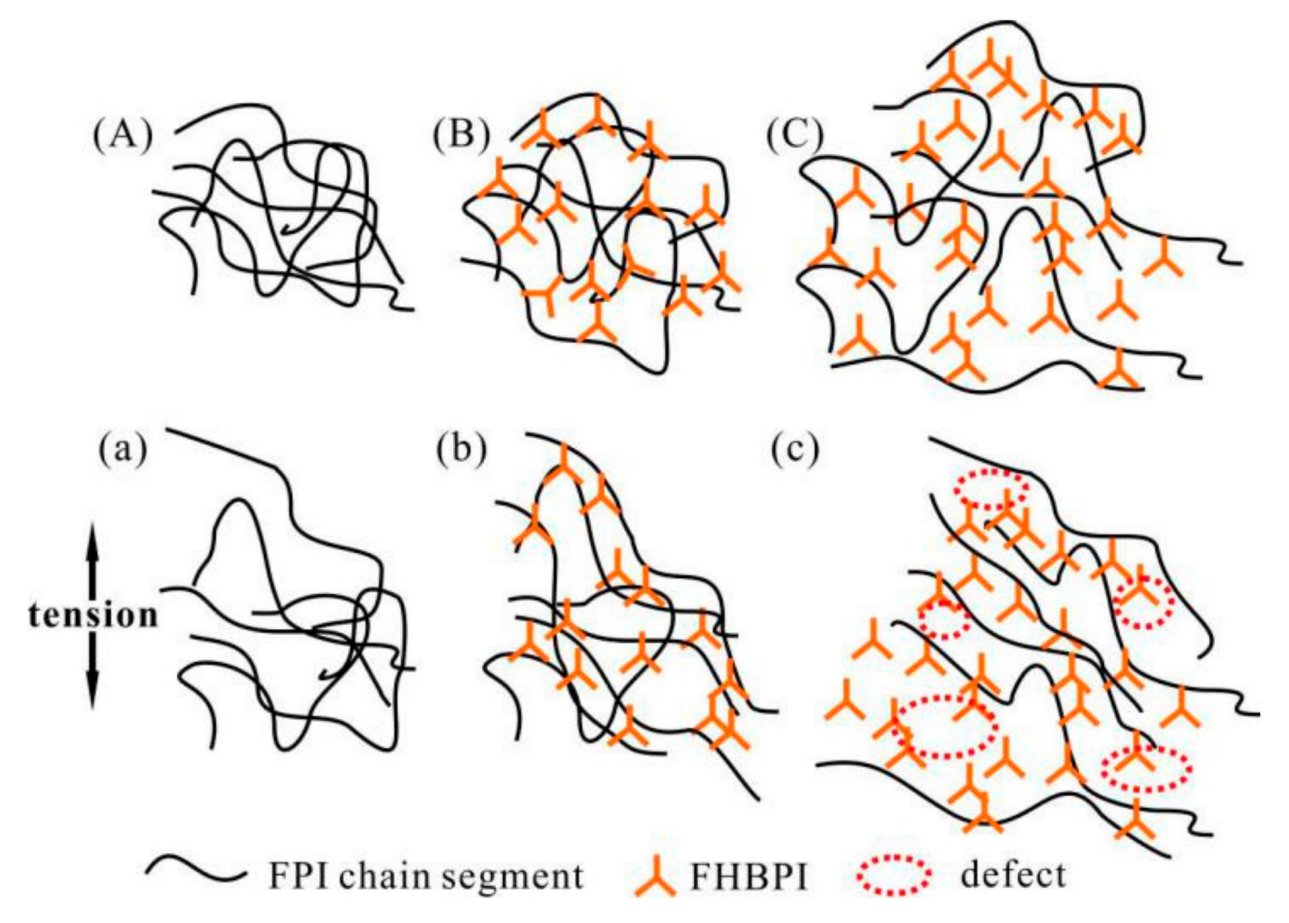
The conceptional pictures of FHBPI distributed in FPI matrix: (**A**) Pure FPI, (**B**) Homogeneous PI/FHBPI physical cross-linking networks at low FHBPI content, (**C**) Aggregated FHBPI molecules block physical cross-linking of FPI molecular chains at high FHBPI content. The (**a**–**c**) are pictured after the tension, respectively.

In our present work, we identified that low content of FHBPI (≤15 wt %) could appropriately increase the distance and act as physical cross-linking points between FPI molecular chains. However, excessive FHBPI (>15 wt %) will flood the FPI molecular chains and greatly increase FPIs’ intermolecular distance, indicating that intermolecular forces are extremely declining. Moreover, there is a lack of chain entanglements between FHBPI molecules [44]. According to the above two points, excessive FHBPI will harm the mechanical properties of the FPI/FHBPI composites (Figure 4). Concretely, only compact physical entanglements between chains are present in pure FPI (Figure 4A). When low levels of FHBPI is replenished, it can act as physical cross-linking points between the FPI chains, also increasing their distances, due to hyperbranched bulk structure (Figure 4B). When stress is applied, the bond length and bond angle of the FPI molecular chains begin to change. More stress is transferred to the physical cross-linking point (FHBPI) with an increase of external pull, resulting in the extension of FPI molecular segments in the direction of tension but no mutual slippage (Figure 4b). Therefore, the formed network can burden much more stress. Nevertheless, FHBPI molecules are relatively rigid because a large number of benzene rings exist in their molecular structure. Hence, the FPI/FHBPI composites exhibit strong and rigid mechanical properties with the addition of FHBPI. The elongation at break of FPI/FHBPI composites is, thus, gradually reduced, again verifying the results of the tensile test. When high levels of FHBPI is added, effective chain entanglements will be reduced between FPI molecular chains owing to the blockage of numerous large-volume FHBPI molecules. FHBPI molecules also lack chain entanglements between themselves (Figure 4C). However, a weak site usually occurs where there is a lack of chain entanglement when stress spreads in the composite materials. Stress tended to concentrate on defects, immediately resulting in the fracture of FPI/FHBPI composites (Figure 4c). Furthermore, because a small amount of FHBPI had effective physical entanglements with FPI molecular chains and acted as insurmountable obstacles, the propagating cracks can be prevented. This was why FHBPI improved the mechanical strength of the whole system [45].

**Figure 5:**
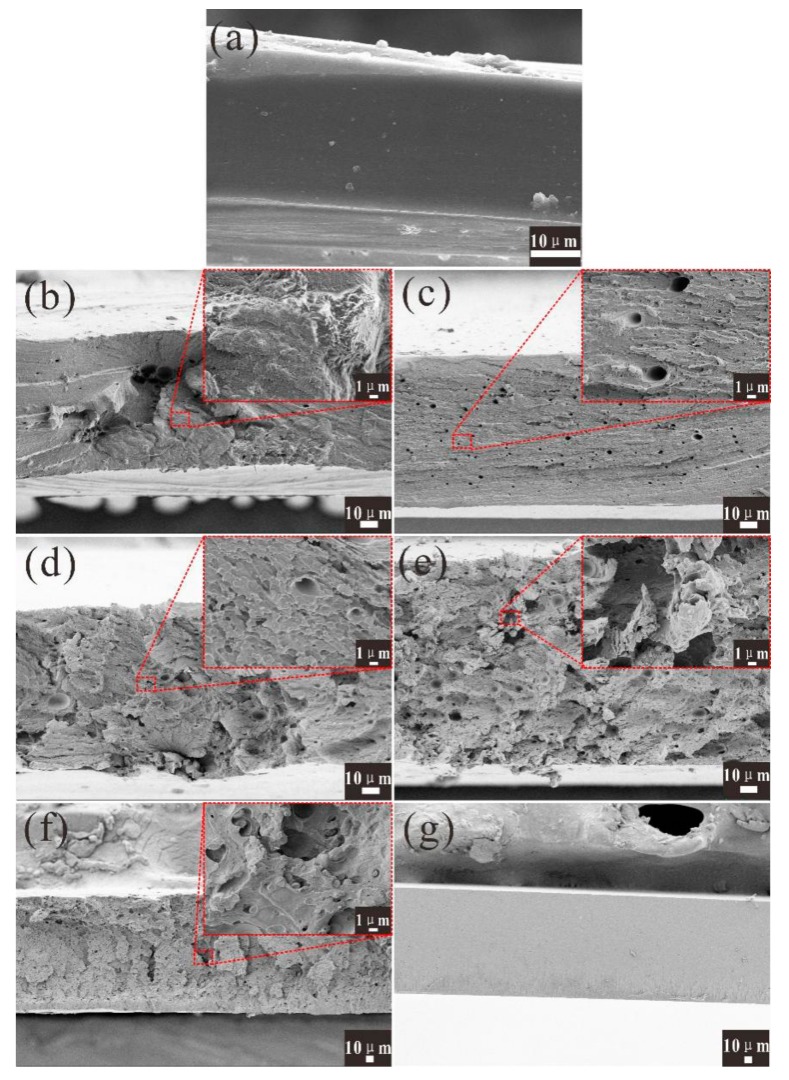
FESEM fractographs of FPI, FPI/FHBPI composites and FHBPI. (**a**) Pure FPI, (**b**) FPI/FHBPI-5%, (**c**) FPI/FHBPI-10%, (**d**) FPI/FHBPI-15%, (**e**) FPI/FHBPI-20%, (**f**) FPI/FHBPI-25%, (**g**) FHBPI.

The change of microstructure can also reflect the change of mechanical properties of materials. The FPI film had a relatively smooth fracture surface with only some protuberances resulting from its shrinkage deformation (Figure 5a). With the addition of only 5 wt % FHBPI, the sectional appearance of FPI/FHBPI-5% was noticeably different (Figure 5b). Its cross-section became rough with more plastic deformed veins. More uniform plastic deformed veins appeared on the section of FPI/FHBPI-10%, indicating that plastic deformation still existed and tensile strength was significantly improved (Figure 5c). Attractively, when the content of FHBPI increased to 15 wt %, FPI/FHBPI composite owned a much fluffier cross section. In theory, the addition of FHBPI affects the FPI chain arrangements and bring about increased free volume fraction of the system. Moreover, the propagation of the crack will be prevented by FHBPI, yielding many fluffy, loosen, and void fragments as showed in the cross section of FPI/FHBPI-15%. Continuing to add FHBPI (20 wt %) caused the appearance of more loose deformation textures and voids on the section, as shown in the enlarged view in Figure 5e, indicating that the mechanical properties had started to decline compared to FPI/FHBPI-15%. The change was mainly due to the reduction in the number of chain entanglements in the composite. Uneven textures and larger voids appeared in the fracture surface of FPI/FHBPI-25%, indicating a further decline in mechanical properties. Due to the relatively high rigidity, the section of FHBPI was very flat, reflecting the fragile nature of FHBPI. In summary, low levels of FHBPI could increase chain entanglements with FPI and a stable crosslinking network system was formed. In pure FPI and FHBPI, the relatively plentiful entanglements highly restricted the cracks, resulting in a smooth fracture surface. At low FHBPI content, the physical entanglements within the FPI chains were reduced, due to a large volume structure of FHBPI. The existence of free volume was the key factor of crack generation. Wherefore, a large number of veins were evenly distributed over the section. Nevertheless, at high FHBPI loading, uneven textures and larger voids appeared. This result can be owing to a high concentration of FHBPI, which blocked effective entanglements between FPI molecular chains and resulted in the formation of defects.

### 3.5. Optical Properties

**Figure 6:**
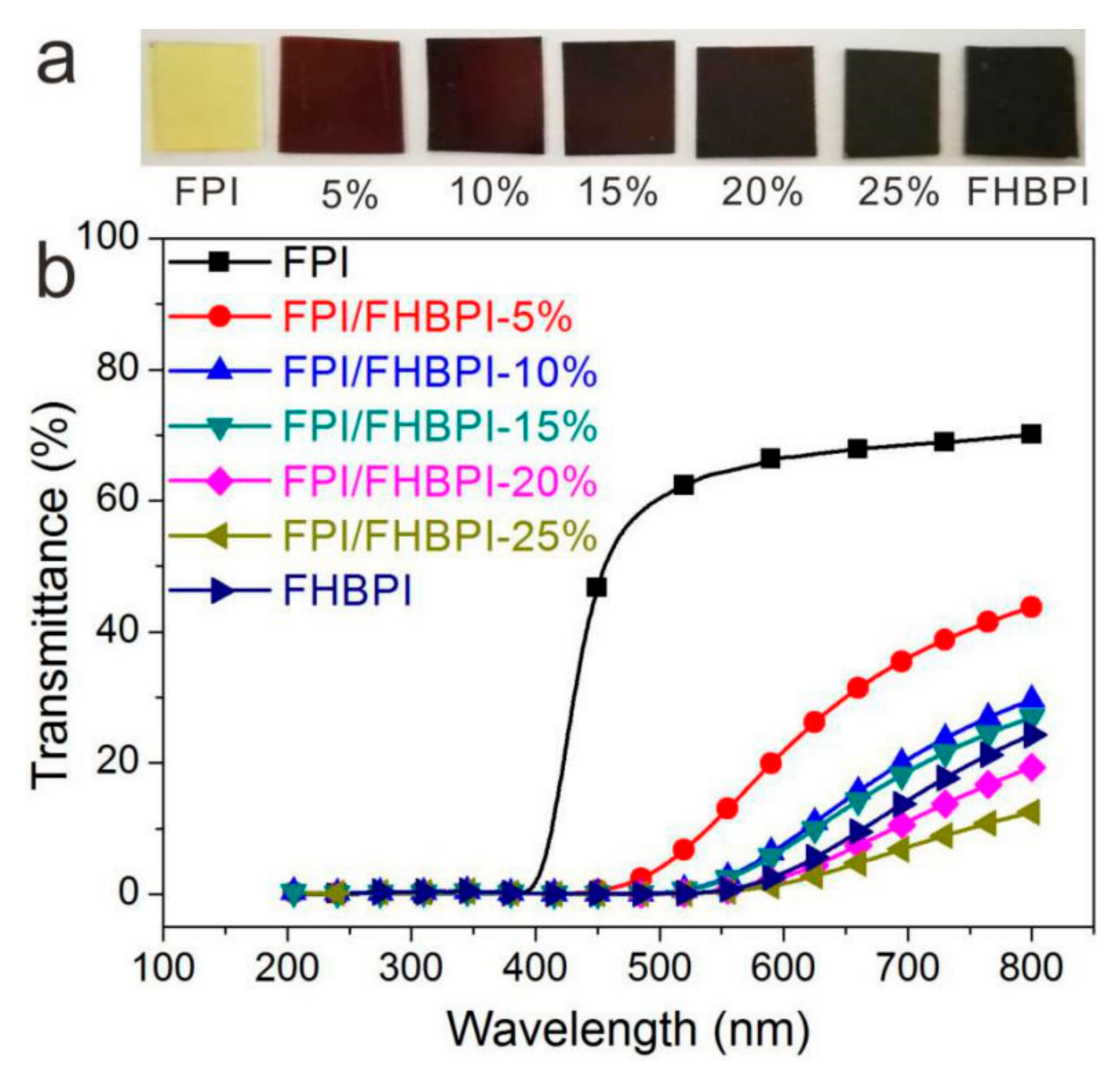
(**a**) Digital photographs and (**b**) UV-vis light transmittance spectra of FPI, FPI/FHBPI composites and FHBPI.

Optical properties are the properties that must be considered when films are used as photoelectric materials. All films prepared in this experiment are relatively uniform in thickness (0.20 ± 0.02 mm). The digital photographs of the FPI/FHBPI composites are shown in Figure 6a. Pure FPI film exhibited a pale-yellow color and high transparency. With the addition of FHBPI into the FPI, the FPI/FHBPI composite films turned from pale-yellow to wine red first and then reddish black, showing decreased transmittance. This was attributed to numerous charge transfer complexes (CTC) within or between molecules of FHBPI, which was brought about by the benzene rings in the molecules. Because CTC absorbs less red light, FPI/FHBPI composite films with low content of FHBPI will reflect the red light into the human eyes, that is, they present wine red. When the content of FHBPI is sufficient, the composite films show reddish black. The UV-vis spectra give the same changes in the wavelengths from 200 to 800 nm (Figure 6b). The FPI film, which exhibited the best optical transmission, only showed 70.1% transmittance at 800 nm. Larger thickness resulted in relatively lower transmittance compared to previously reported pure PI film [33]. The light transmittance of the composites decreased with increasing FHBPI content, mainly attributed to the increased CTC in FPI/FHBPI composites. Interestingly, the transmittance of pure FHBPI film at 800 nm was superior to those of FPI/FHBPI-20% and FPI/FHBPI-25%. This was mainly giving credit to the lack of chain entanglement between the FHBPI molecules, resulting in a large number of free volumes. These free volumes improved the probability of light passing through the film, so the transmission of FHBPI increased. In addition, all of the films possess prominent ultraviolet absorption from Figure 6b, especially for FPI/FHBPI composites. Concretely, the cutoff wavelength of pure FPI film reached 390 nm, which covered 95% of the UV region. By contrast, the truncation wavelength of FPI/FHBPI-5% increased to 420 nm. Moreover, the truncation wavelength of FPI/FHBPI composites continued to increase with increasing FHBPI content. These composites could cover the whole UV region, indicating that they had potential applications in ultraviolet shielding.

### 3.6. UV-Shielding Performance

**Figure 7:**
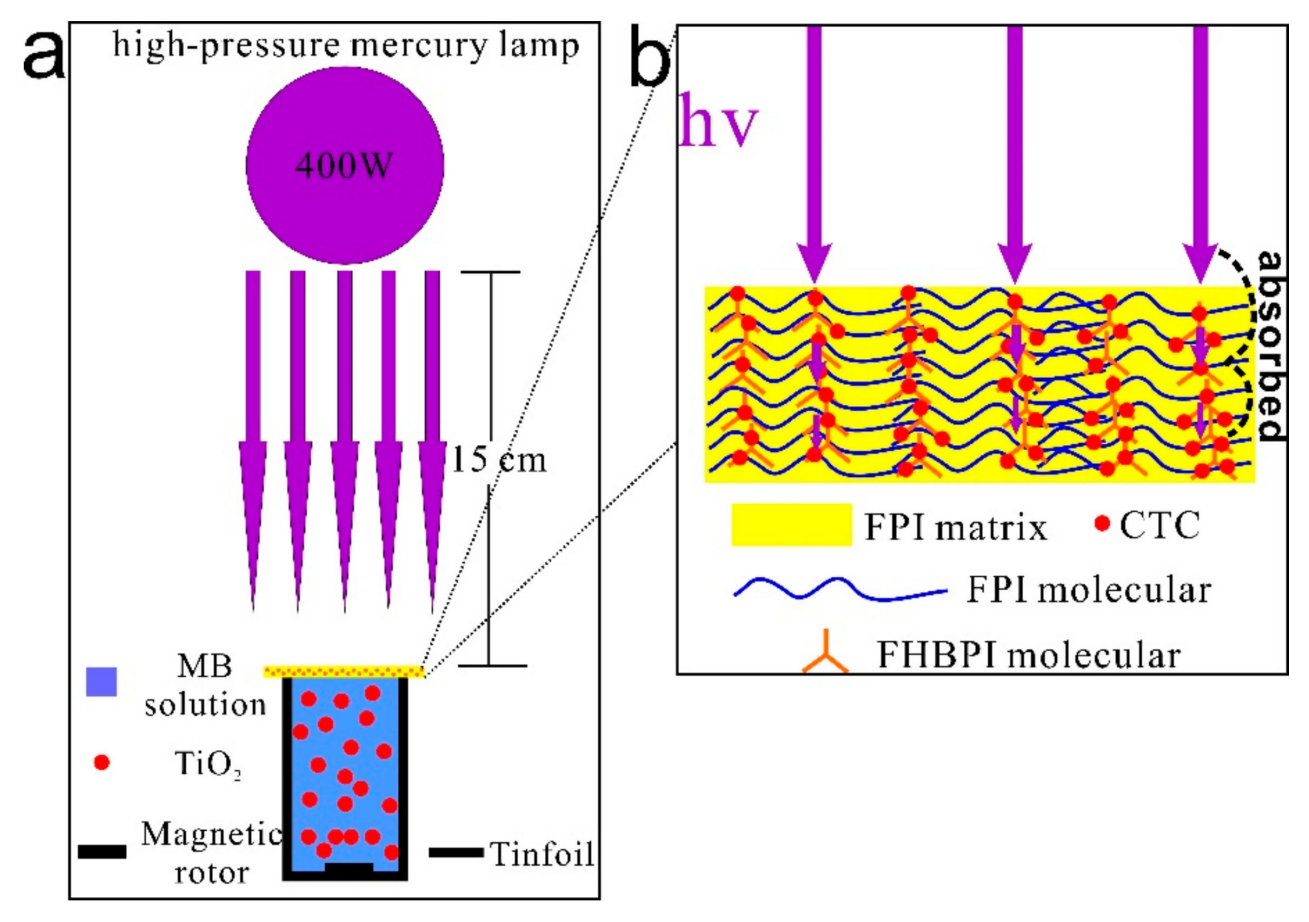
(**a**) Simulation illustration for UV-shielding measurement of FPI/FHBPI composites under intense ultraviolet lights; (**b**) Schematic illustration for UV-shielding of FPI/FHBPI composites.

We designed an experiment for photocatalytic degradation of methylene blue (MB) and then used the prepared FPI/FHBPI composite films to shield the ultraviolet light, monitoring changes in the concentration of MB [7,33], as illustrated in Figure 7a.

**Figure 8:**
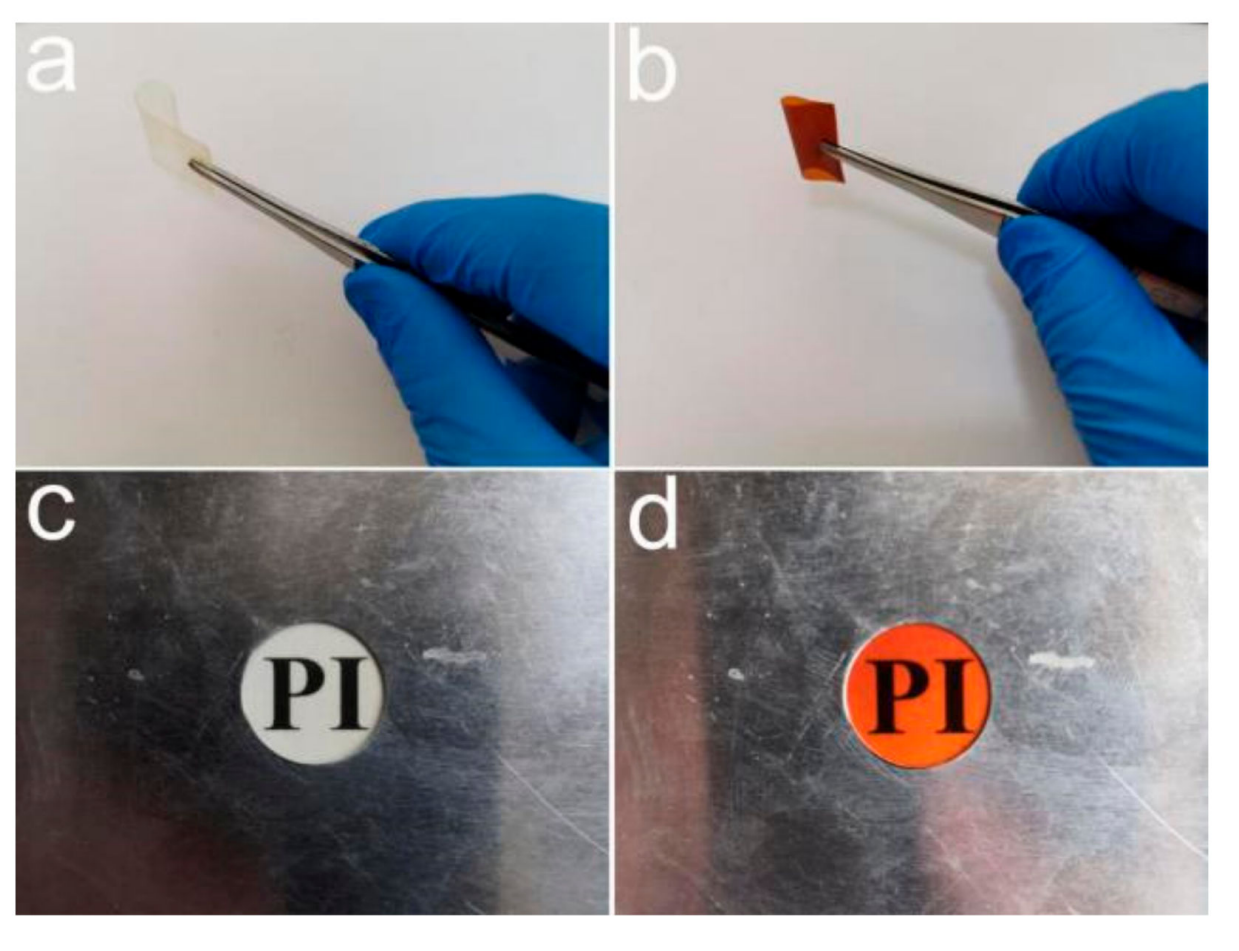
Digital photographs of FPI (**a**,**c**) and FPI/FHBPI-15% (**b**,**d**) (about 0.05 mm thick).

**Figure 9:**
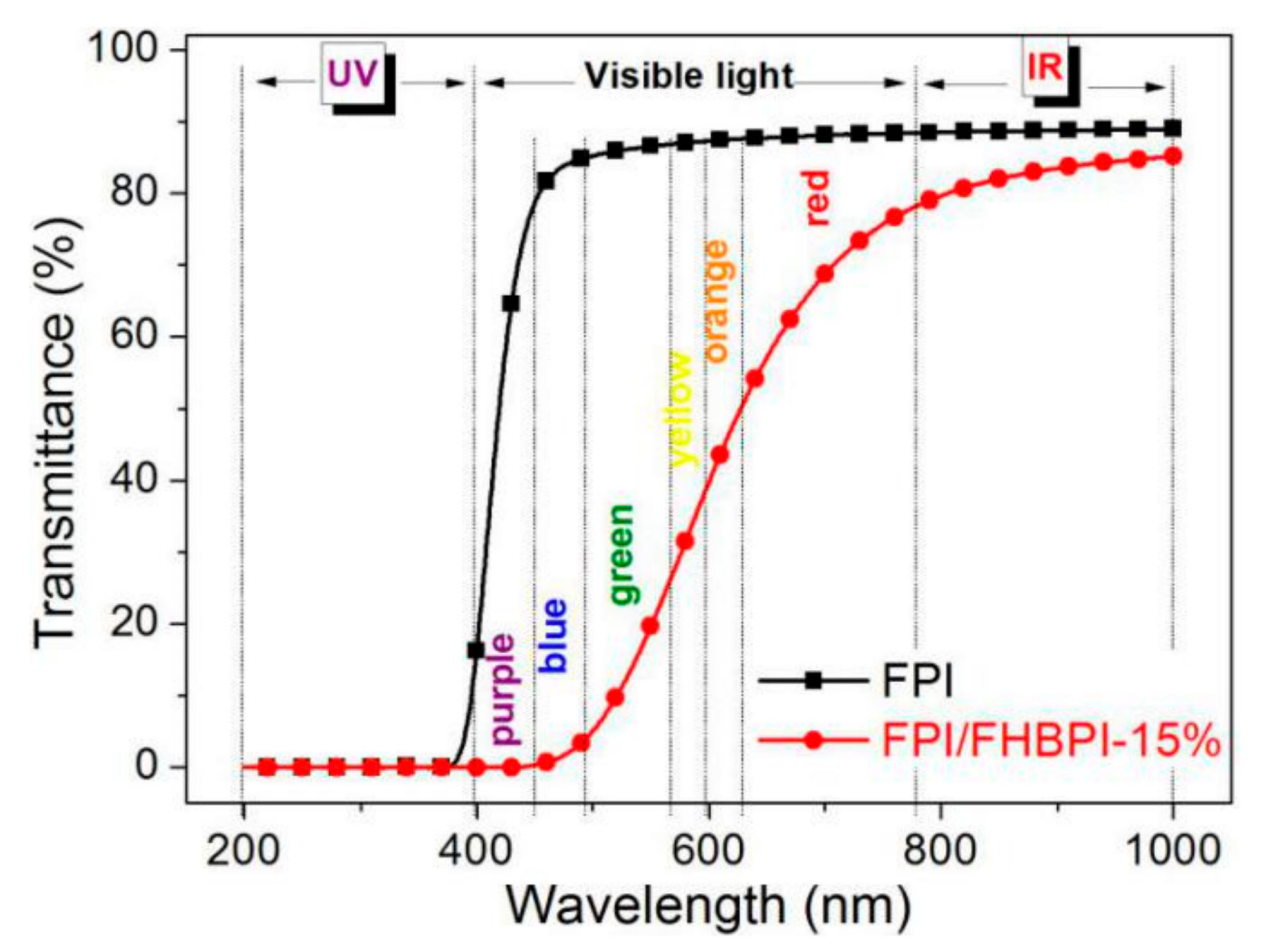
UV-vis light transmittance spectra of FPI and FPI/FHBPI-15% (about 0.05 mm thick).

However, outstanding UV absorption is not the only factor for UV-shielding materials. Excellent transparency to visible light is also essential. It was worth noting that the films prepared in this experiment were too thick, resulting in poor transmission of visible light. Therefore, in order to increase the transmittance of the films to visible light, the approach we take was to reduce the thickness of the films. Take pure FPI and FPI/FHBPI-15% for example, the preparation process was basically the same as the above experimental section, but the only difference was that an equal amount of solution was poured onto a larger mold. According to such method, the prepared films had smaller thicknesses (about 0.05 mm), real product photos, as shown in Figure 8. By attaching the two films to the white paper with the word “PI”, it can be seen that the pure FPI and FPI/FHBPI-15% are pale yellow and orange red, respectively. The word “PI” can be seen clearly, indicating their outstanding transmittance of visible light. Figure 9 gives their transmittance in the wavelengths from 200 to 1000 nm. The region can be divided into three regions: Ultraviolet (UV, 200–400 nm), visible light (400–780 nm), and infrared (IR, 780–1000 nm). Among them, the visible light region can also be divided into purple, blue, green, yellow, orange, and red. As can be seen from the Figure 9, the transmittance of the FPI film at 800 nm is as high as 88.5%, which fully proves that the transmittance of the film is related to its thickness. Furthermore, its transmittance has little loss in most visible light regions, including yellow, orange, and red. In other words, the FPI film has substantially no absorption of these three kinds of light. Moreover, the smaller the wavelength, the more easily the light is reflected. Therefore, the FPI film seen by the human eyes is yellow. FPI/FHBPI-15% film has a relatively high transmittance for orange and red. Therefore, by the same token, it shows orange-red. At the same time, we can notice that the transmittance of the FPI/FHBPI-15% film at 800 nm still retains 79.6%. This indicates that the FPI/FHBPI-15% film still has a good transmittance in the visible light region, which can meet the basic requirements as an ultraviolet shielding material.

**Figure 10:**
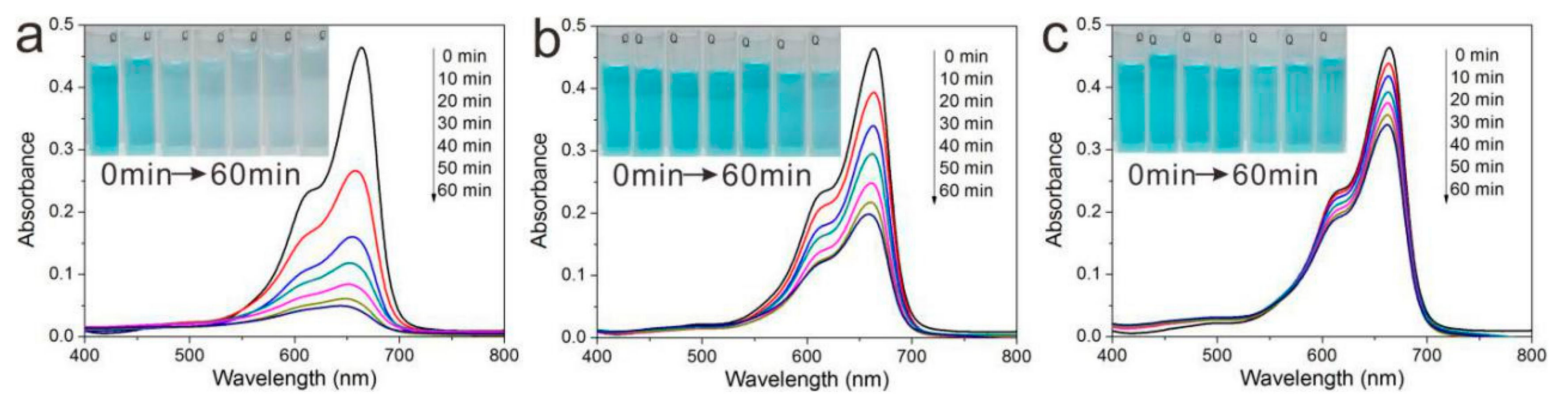
Time-dependent UV-vis spectra for the photodegradation of MB in the presence of TiO_2_ nanoparticles under no protection (**a**) and protected by FPI with 0.05 mm thick (**b**), FPI/FHBPI-15% with 0.05 mm thick (**c**). The inserted photographs show the corresponding changes in MB solutions in color.

**Figure 11:**
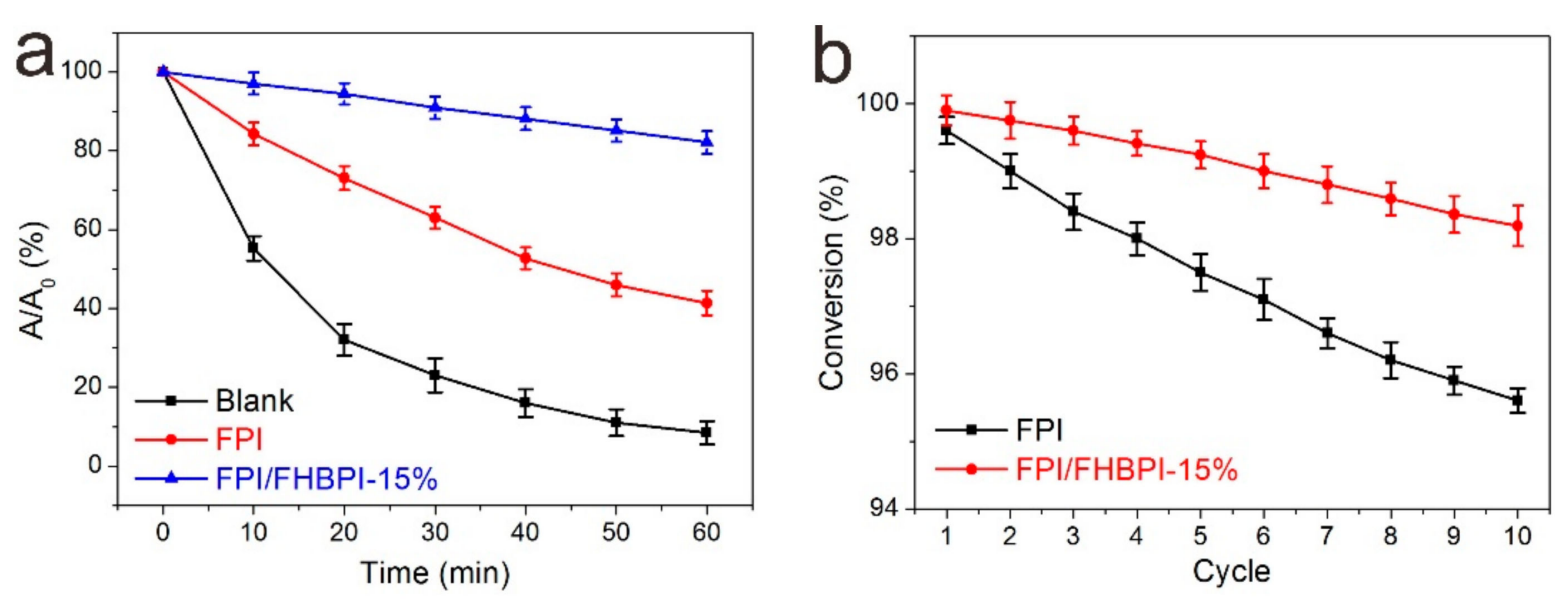
(**a**) Decay curves of the absorption intensity of the MB solution at 665 nm under no protection and protected by FPI and FPI/FHBPI-15% with 0.05 mm thick. (**b**) Recyclability of FPI and FPI/FHBPI-15% with 0.05 mm thick as UV-shielding materials for MB solution.

The UV characteristic absorption peak of MB is at 665 nm, so the shielding efficiency of the films can be calculated by monitoring the UV absorption of MB solution at 665 nm. In a control study (Figure 10a), the absorption intensity of MB was gradually decreasing until substantially complete degradation after 60 min of UV irradiation. The inserted photos represented MB solution at each time point and showed changes in the color. The MB solution in the control study changed from blue to colorless, indicating its effective degradation directly under intense ultraviolet ray (400 W) in the case of nano-TiO_2_ as a catalyst. By contrast, the MB solution was only partially degraded under cover of pure FPI film, and 41.4% of MB was retained (Figure 10b). Its color also changed from blue to light blue. When covered by FPI/FHBPI-15 wt %, the MB solution at 665 nm showed a smaller drop in absorption intensity (Figure 10c). Moreover, 82.2% of MB was still retained, and its color changed to a lesser extent. In order to quantitatively evaluate the masking properties of the two films, the attenuation curve was plotted for the absorption intensities of the MB solutions at 665 nm (Figure 11a). Obviously, the pure FPI owned a certain degree of UV-shielding performance, since there were still 41.4% MB retained. When 15 wt % FHBPI was added to the FPI, the rate at which MB was degraded was significantly reduced under the shadow of the obtained film. Only 17.8% of the MB was degraded, indicating that thinner FPI/FHBPI composite films still had outstanding UV-shielding performance under intense UV radiation. The improvement was ascribed to the CTCs, brought by FPI and FHBPI. Intermolecular and intramolecular CTCs endow FPI with UV absorption capacity. Moreover, FHBPI molecule contains more benzene rings, resulting in the formation of more conjugated structures. Therefore, more CTCs are formed. In addition, when FHBPI was added, not only does the CTCs brought by itself be introduced into the composite system, but also new CTCs are formed between FHBPI with FPI molecular chains. The UV-shielding mechanism of the FPI/FHBPI composite films is shown in Figure 7b. Due to the introduction of FHBPI, the ultraviolet absorption capacities of the FPI/FHBPI composites are obviously enhanced compared to the reported pure PI matrix [38]. FPI/FHBPI composites are better PI substrates if we want to further enhance the ultraviolet absorption capacities of PI by adding fillers. Furthermore, through the characterization of the above thermal properties, we found that the FPI/FHBPI-15% had excellent thermal properties (*T*_d,5%_: 505.7 °C in N_2_; *T*_g_: 246.7 °C). Other reported UV-shielding materials were less stable than our prepared materials, such as polyvinyl alcohol (PVA) and polyurethane (PU) [46,47,48]. In the near future, the prepared FPI/FHBPI-15% may be applied as an UV-shielding material in some harsh environment, such as high temperature, intensive UV, etc.

Except for UV-shielding efficiency, recyclability is equally important for UV-shielding materials [49,50,51,52,53]. To investigate the recyclability of the pure FPI film and FPI/FHBPI-15%, the repeated trials were designed. The FPI/FHBPI-15% still maintained high UV-shielding activity after 10 cycles with 98.19% of the conversion (Figure 11b). Hence, the prepared FPI/FHBPI composites were potential recyclable UV-shielding materials.

### 3.7. Surface Contact Angle (SCA), and Water Absorption (WA)

**Figure 12:**
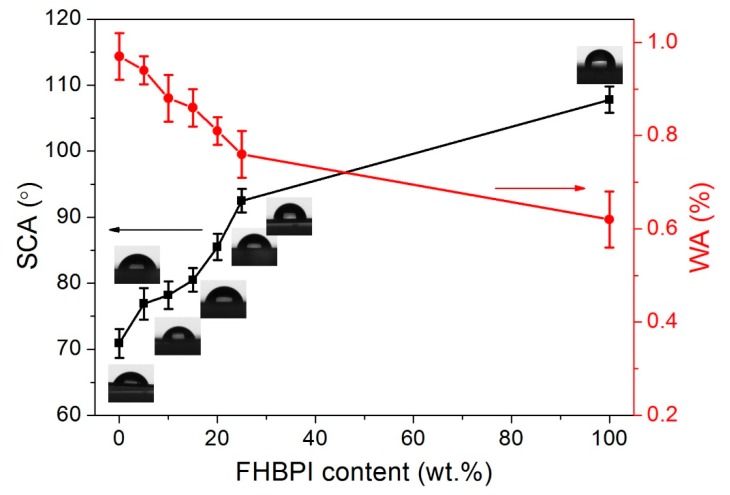
Effects of FHBPI content on *SCA* and *WA* of FPI composites.

The *SCA* and *WA* of PIs have a great influence on their photoelectric stability [54,55,56,57]. Low *WA* and high *SCA* can reduce their dielectric constants and delay corrosion of electric wires in integrated circuits. Hence, low *WA* and relatively high *SCA* are extremely necessary for PIs. The *SCA* and *WA* of the FPI/FHBPI composites are shown in Table 3. These values change regularly as the FHBPI content increases, summarized in Figure 12. It can be seen that the *SCA* of the pure FPI is just 70.9°, which is probably attributed to numerous ether linkages in its structure. With the increase of FHBPI content, the FPI/FHBPI composites exhibit increased *SCA*. FPI/FHBPI-25% can obtain an SCA of 92.5°, 21.6° more than the pure FPI film. In addition, the *SCA* of FHBPI is up to 107.8°, indicating that the FHBPI molecule contains more hydrophobic benzene rings than hydrophilic ether bonds. This is also the reason why the *SCA* of FPI/FHBPI composites increases with the increase of FHBPI content. Similarly, it can also be seen from Figure 12 that the *WA* of FPI/FHBPI composites decreases with the increase of FHBPI content. This trend is also caused by a relative decrease in the water-absorbing ether bond in the FHBPI molecule. In general, the addition of FHBPI reduces *WA* and increases *SCA* of the composite system, guaranteeing stability as dielectric materials in electronic circuits.

## 4. Conclusions

In this article, a series of FPI/FHBPI composites were successfully prepared by the simple blend of FPI and FHBPAA, and subsequently thermal imidization. FPI possessed superior compatibility with FHBPI, and no obvious phase separation was found. The composites exhibited improved mechanical, UV-shielding, and hydrophobic properties compared to the pure FPI film. The incorporation of FHBPI led to the formation of a physical crosslinked network structure between FPI and FHBPI, and significantly enhanced the mechanical strength of FPI. The maximum increase in tensile strength reached 83% when 15 wt % of FHBPI was added. Moreover, the FPI/FHBPI-15% owned remarkable UV-shielding property and lifetime under intense UV ray. In consideration of the composites’ superior heat stability and mechanical property, FPI/FHBPI composites may be applied as UV-shielding materials in some harsh environment, such as high temperature, intensive UV, etc. Specifically, they may be used to coat the glass in spacesuits and capsules to block out UV rays in the near future. Alternatively, the flag can be encapsulated into the FPI/FHBPI composite films so that it can stay in space for a long time without fading.

## Figures and Tables

**Table 1:** Thermal properties of FPI, FPI/FHBPI composites and FHBPI.

Sample	*T*_g_ (°C) ^a^	*T*_d_ (°C)		*R*_w_ (wt %) ^b^
		*T*_d,5%_	*T*_d,10%_	
FPI	260.3	523.2	542.1	59.9
FPI/FHBPI-5%	264.5	510.4	530.1	59.3
FPI/FHBPI-10%	255.8	508.1	528.7	57.8
FPI/FHBPI-15%	246.7	505.7	524.4	58.6
FPI/FHBPI-20%	235.4	501.3	519.7	58.0
FPI/FHBPI-25%	246.7	498.3	516.2	56.3
FHBPI	261.9	485.3	514.6	54.5

^a^ Glass-transition temperatures by DMA. ^b^ Residue weight fraction at 800 °C in N_2_.

**Table 2:** Tensile strength and elongation at break of FPI, FPI/FHBPI composites and FHBPI.

Sample	Tensile Strength (MPa)	Elongation at Break (%)
FPI	71.7 ± 2.3	11.14 ± 1.05
FPI/FHBPI-5%	92.4 ± 3.2	9.67 ± 1.24
FPI/FHBPI-10%	123.0 ± 2.6	9.10 ± 1.11
FPI/FHBPI-15%	131.4 ± 3.1	8.26 ± 0.96
FPI/FHBPI-20%	107.8 ± 2.9	7.91 ± 1.28
FPI/FHBPI-25%	64.38 ± 3.7	6.16 ± 1.35
FHBPI	66.9 ± 3.9	4.60 ± 1.22

**Table 3:** Surface contact angle (*SCA*) and *WA* of FPI, FPI/FHBPI composites and FHBPI.

Sample	*SCA* (°)	*WA* (%)
FPI	70.9 ± 2.2	0.97 ± 0.05
FPI/FHBPI-5%	76.9 ± 2.4	0.94 ± 0.03
FPI/FHBPI-10%	78.2 ± 2.1	0.88 ± 0.05
FPI/FHBPI-15%	80.5 ± 1.8	0.86 ± 0.04
FPI/FHBPI-20%	85.5 ± 2.0	0.81 ± 0.03
FPI/FHBPI-25%	92.5 ± 1.8	0.76 ± 0.05
FHBPI	107.8 ± 2.0	0.62 ± 0.06
